# Identification of wheat seedling varieties based on MssiapNet

**DOI:** 10.3389/fpls.2023.1335194

**Published:** 2024-01-18

**Authors:** Yongqiang Feng, Chengzhong Liu, Junying Han, Qinglin Lu, Xue Xing

**Affiliations:** ^1^ College of Information Sciences and Technology, Gansu Agricultural University, Lanzhou, China; ^2^ Wheat Research Institute, Gansu Academy of Agricultural Sciences, Academy of Agricultural Sciences, Lanzhou, China

**Keywords:** wheat, seedlings, variety identification, scse attention, visualized, feature fusion

## Abstract

**Introduction:**

In the actual planting of wheat, there are often shortages of seedlings and broken seedlings on long ridges in the field, thus affecting grain yield and indirectly causing economic losses. Variety identification of wheat seedlings using physical methods timeliness and is unsuitable for universal dissemination. Recognition of wheat seedling varieties using deep learning models has high timeliness and accuracy, but fewer researchers exist. Therefore, in this paper, a lightweight wheat seedling variety recognition model, MssiapNet, is proposed.

**Methods:**

The model is based on the MobileVit-XS and increases the model's sensitivity to subtle differences between different varieties by introducing the scSE attention mechanism in the MV2 module, so the recognition accuracy is improved. In addition, this paper proposes the IAP module to fuse the identified feature information. Subsequently, training was performed on a self-constructed real dataset, which included 29,020 photographs of wheat seedlings of 29 varieties.

**Results:**

The recognition accuracy of this model is 96.85%, which is higher than the other nine mainstream classification models. Although it is only 0.06 higher than the Resnet34 model, the number of parameters is only 1/3 of that. The number of parameters required for MssiapNet is 29.70MB, and the single image Execution time and the single image Delay time are 0.16s and 0.05s. The MssiapNet was visualized, and the heat map showed that the model was superior for wheat seedling variety identification compared with MobileVit-XS.

**Discussion:**

The proposed model has a good recognition effect on wheat seedling varieties and uses a few parameters with fast inference speed, which makes it easy to be subsequently deployed on mobile terminals for practical performance testing.

## 1 Introduction

Wheat, one of the three major staple grains, is essential in ensuring food security and stabilizing the country’s economic development (Kiss, 2011). China is a predominantly agricultural country, a populous country, a global wheat producer and consumer (He, 2001), and to maximize yields, growers choose the right combination of varietal duration and sowing time to ensure that the crop blooms at the optimum period (Liu et al., 2023). However, in the actual planting, affected by environmental factors, some fields will appear to lack seedlings; when this situation occurs, it is necessary to use the same variety of wheat to make up for the lack of seedlings to avoid the reduction of wheat yield (Zhu, 2021).

Traditionally, there are many ways to identify wheat seedling varieties, and one of the most reliable methods is the molecular identification of wheat varieties (Gupta et al., 1999). However, the timeliness of detection using this method is poor and not suitable for real-time classification detection. With the development of science and technology, deep learning techniques are increasingly used in wheat variety identification and disease detection. Long M et al (Long et al., 2023). proposed CerealConv classification model for healthy plants and four foliar diseases of wheat, yellow rust, brown rust, powdery mildew, and seven-leaf spot with 97.05% classification accuracy. Zhao X et al (Zhao et al., 2022). proposed a hybrid convolutional network based on hyperspectral images to classify wheat seed varieties with an accuracy of 95.65%. Goyal L et al (Goyal et al., 2021). used an improved deep learning approach to classify ten diseases of wheat spikes and leaves with a final accuracy of 97.88%. Velumani K et al (Velumani et al., 2020). used a convolutional neural network to predict the date of wheat tasselling and compared the results obtained with the actual date of tasselling, finding it to be more effective.

These studies have shown promising results in disease identification, seed variety classification, and tassel date prediction in wheat, but fewer studies use convolutional neural networks to classify grain at the seedling stage. This study aimed to classify wheat seedlings using the improved MobileVIT-XS method to aid identification.

Before VIT (Dosovitskiy et al., 2020), self-attention (Vaswani et al., 2017) had limited application in CV, used with convolution or by replacing specific modules inside a CNN with self-attention, but the overall architecture remained the same. The emergence of VIT shows that it is possible to get good results in image classification tasks using only a simple Vision Transformer structure, opening a new era of CV. VIT networks still have problems, such as a high number of parameters and slow inference speed compared with traditional CNN networks in practical applications. MobileViT (Mehta and Rastegari, 2021) is mainly designed and proposed to solve the defects of the ViT network, incorporating the advantages of CNN into the structure of the Transformer to solve the shortcomings of the Transformer network, such as difficulty in training, migration, and adjustment, and to accelerate the inference and convergence speed of the network, which makes the network more stable and efficient.

MobileViT has been applied to agriculture by more and more researchers due to its lightweight network structure and high recognition accuracy. Sheng X et al (Sheng et al., 2022). used a MobileVit-based convolutional block to replace the traditional convolutional block for feature extraction, thus proposing a cascaded backbone network for fruit tree leaf disease recognition with an accuracy of 96.76. Long C et al (Long et al., 2023). used MobileViT_v2 as a backbone network to identify wild mushroom species by introducing CA (Hou et al., 2021) blocks and introducing jump connections between the blocks, and the classification accuracy of the obtained Top1 was 97.39%. Liu Z et al (Liu et al., 2022a). used KNN (Cover and Hart, 1967), SVM (Schölkopf and Smola, 2002), and MobileViT-xs methods to identify moldy peanuts with improved accuracy of 3.55%, 4.42%, and 5.9%. Sun Q et al (Sun et al., 2022). proposed SADNet to segment orchard UAV images by introducing DIC, ASPP, and scSE modules with a final pixel accuracy of 93.61%. Deng H et al (Deng et al., 2022). achieved a final segmentation model PA of 95.5% by adding the scSE attention module to the DeepLabv1+ encoder’s backbone network to improve the model’s ability to extract fine features of the paddy field. Dai M et al (Dai et al., 2023). detected chili leaf diseases by optimizing the network depth and width of the Inception module with an accuracy of 97.87%.

The above study improved the MobileVit model to achieve better recognition results for specific targets. In this study, to achieve real-time, highly accurate, and low-cost wheat seedling variety identification by piggybacking the model on a mobile phone and thus, inspired by the Inception (Szegedy et al., 2015) module and the scSE (Roy et al., 2018) module applications in agriculture, the MobileVit-XS model was improved and MssiapNet was proposed. The main contributions of this work include the following:

(1) A new lightweight model for accurately identifying wheat seedling varieties is presented. Based on the MobileVit-XS model, the scSE attention mechanism was first introduced to the MV2 module to improve the sensitivity to the nuances of the wheat seedlings of each variety and suppress the background’s influence on recognition accuracy. Secondly, by improving the Inception V1 module, the IAP module is proposed, which is introduced to perform multi-feature fusion recognition of the results obtained from the MobileVit block to improve the overall recognition accuracy of the model.
(2) To meet the requirements of the data needed in conducting the training, we collected 29,020 wheat seedling images of 29 varieties at the Comprehensive Experimental Station of the National Wheat Industry Technology System in Tianshui City, Gansu Province, China, for evaluating the performance of the model.
(3) The results obtained after fully training the model and loading it onto the test set for recognition show that the model has a high recognition accuracy and a low number of parameters, which is conducive to subsequent deployment on mobile to test in the field.

The structure of this study is as follows: firstly, images of wheat seedlings of different varieties are collected from the field to form an image dataset; secondly, the effectiveness of the proposed MSSE2 module, IAP module, and MssiapNet is verified on the developed dataset; and finally, the experimental results are elaborated.

## 2 Materials and methods

### 2.1 Data set establishment and segmentation

**Figure 1:**
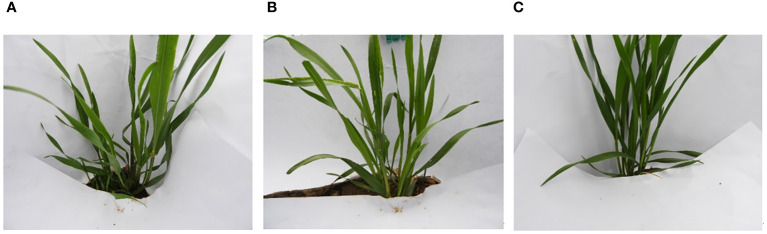
Images of Jimai19 **(A)**, Lantian15 **(B)** and Zhoumai19 **(C)** seedlings.

The wheat seedling image dataset in this experiment was collected from the Comprehensive Experimental Station of the National Wheat Industry Technology System located in Tianshui City (35°44′N, 106°08′E, average elevation 1413 m, average annual rainfall 570 mm, annual sunshine hours 2012 h). Photographed outdoors under natural light conditions with a Nikon COOLPIX B700 digital camera, the format of the captured pictures was JPG. The original images of seedlings of some varieties are shown in **Figure 1**. The 29 wheat varieties selected were all mainstream winter wheat varieties in Gansu Province (Lu Q et al., 2022), such as Jimai 19, Lantian 15, Zhoumai 19, etc. About 30 plants of each variety were selected, and the images were taken at multiple angles. The seedling dataset has 29,020 images. The storage space of a single image in the original image is about 600 kb; the specific information of this dataset is shown in **Table 1**. 90% of the data is selected as the training, and 10% of the data is selected as the test.

**Table 1:** Wheat seedling image dataset.

Name	Classes	Number	Name	Classes	Number	Name	Classes	Number
Jimai19	1	969	Lantian34	11	999	Lantian53	21	972
Jimai20	2	988	Lantian35	12	917	Lantian54	22	973
Jimai21	3	999	Lantian36	13	999	Lantian55	23	597
Jimai22	4	999	Lantian37	14	979	Lantian56	24	997
Jimai44	5	989	Lantian39	15	999	Zhoumai19	25	981
Jimai47	6	999	Lantian40	16	999	Zhoumai20	26	990
Lantian15	7	999	Lantian42	17	968	Zhoumai21	27	999
Lantian19	8	982	Lantian43	18	989	Zhoumai22	28	999
Lantian26	9	979	Lantian45	19	999	Zhoumai23	29	977
Lantian33	10	808	Lantian48	20	977			

### 2.2 Image pre-processing

When performing model training, the dataset first needs to be preprocessed to improve the performance and effectiveness of the model. For the training set, firstly, the given image is randomly cropped to different sizes and aspect ratios using the transforms.RandomResizedCrop(224) method, then the cropped image is scaled to 224X224; secondly, the image is flipped using the transforms.RandomHorizontalFlip() method; finally, transforms.ToTensor() is used to convert the read image to Tensor format, and the converted image is normalized using transforms.Normalize() method. For the test set, the given image is first randomly cropped to different sizes and aspect ratios using the transforms.RandomResizedCrop(224) method, and then the cropped image is scaled to 224X224; finally, the read image is converted to Tensor format using the transforms.ToTensor() and the converted image are normalized using the transforms.Normalize() method. To normalize the training and test images, the mean = [0.57, 0.61, 0.62] and std = [0.21, 0.21, 0.26] were calculated for the entire dataset.

## 3 Our proposed method

### 3.1 scSE Attention Mechanisms

**Figure 2:**
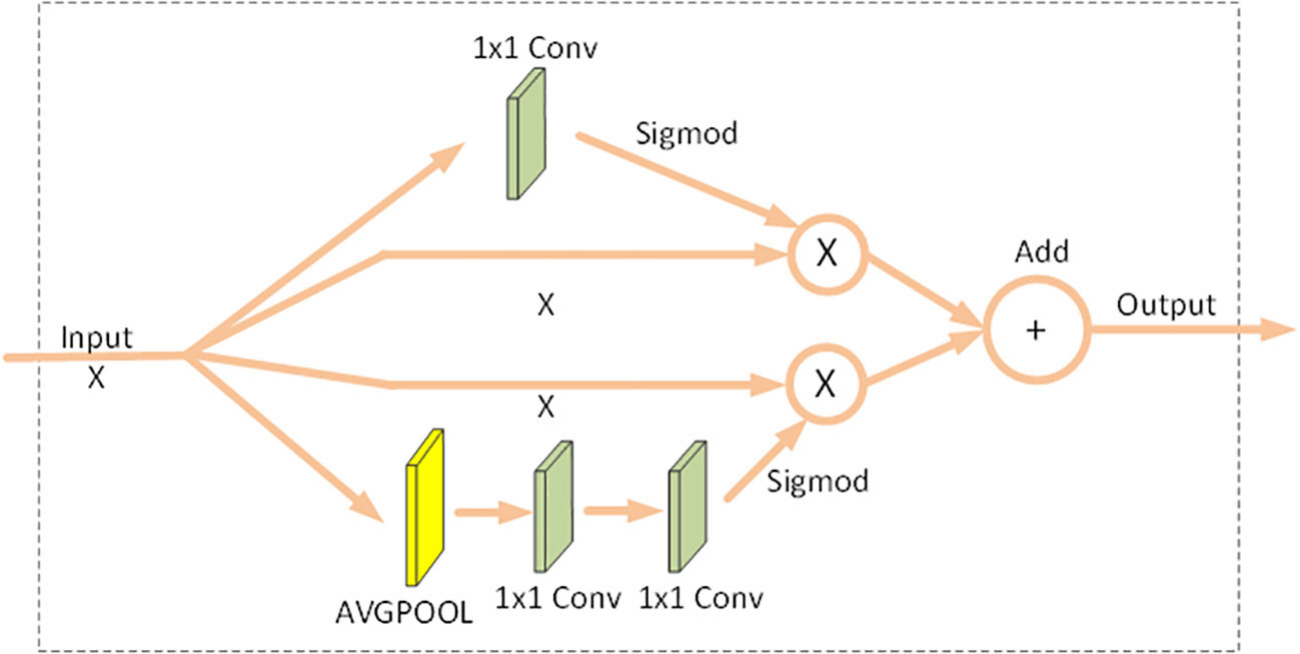
SCSE Attention.

The scSE module is a parallel combination of spatial attention and channel attention, as opposed to the serial form of CBAM. In Spatial Attention: The input data is firstly passed through Conv2d to get b * 1 * h * w structure. Secondly, it goes through sigmoid to amplify focus and suppress non-focus, and finally, the obtained data is multiplied with the original data. In Channeled Attention: Firstly, an adaptive pooling layer is used to get the data in b * ch * 1 * 1 dimensions; secondly, the channels are first Dimensionality Boost and then Dimensionality Reduction, and finally, the weights are obtained using a sigmoid, which is multiplied with the original data to get the channel attention results. The results of spatial attention and channel attention obtained above are summed to form the final result of the scSE module. This attention mechanism is shown in **Figure 2**.

### 3.2 MSSE2 module

**Figure 3:**
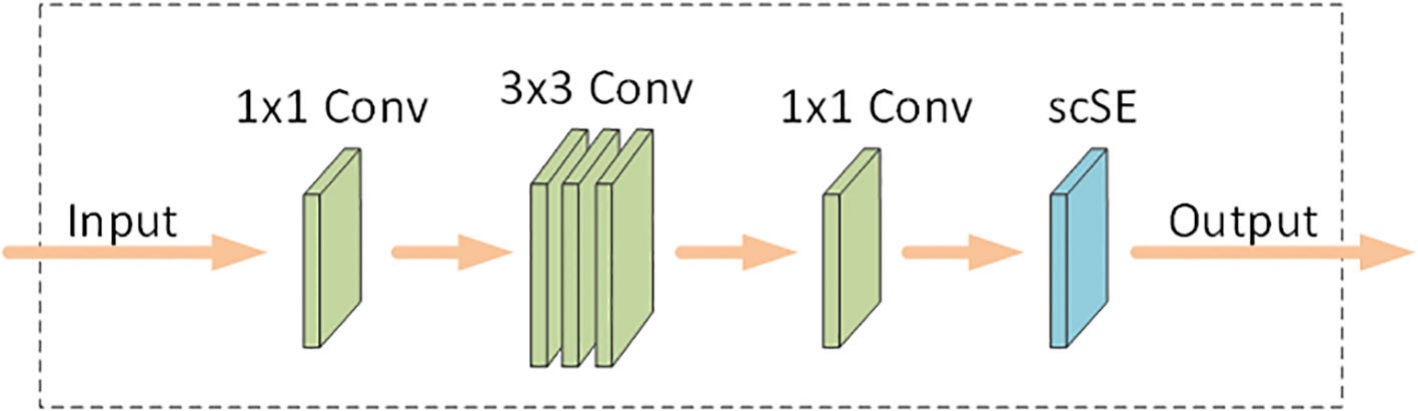
MSSE2 module.

To improve the model’s sensitivity to the subtle differences between seedlings of different wheat varieties and to suppress the influence of the background on the recognition accuracy, the scSE attention module was added to the MV2 module, and the module after the addition of the attention was named MSSE2. The module is shown in **Figure 3**.

### 3.3 IAP module

**Figure 4:**
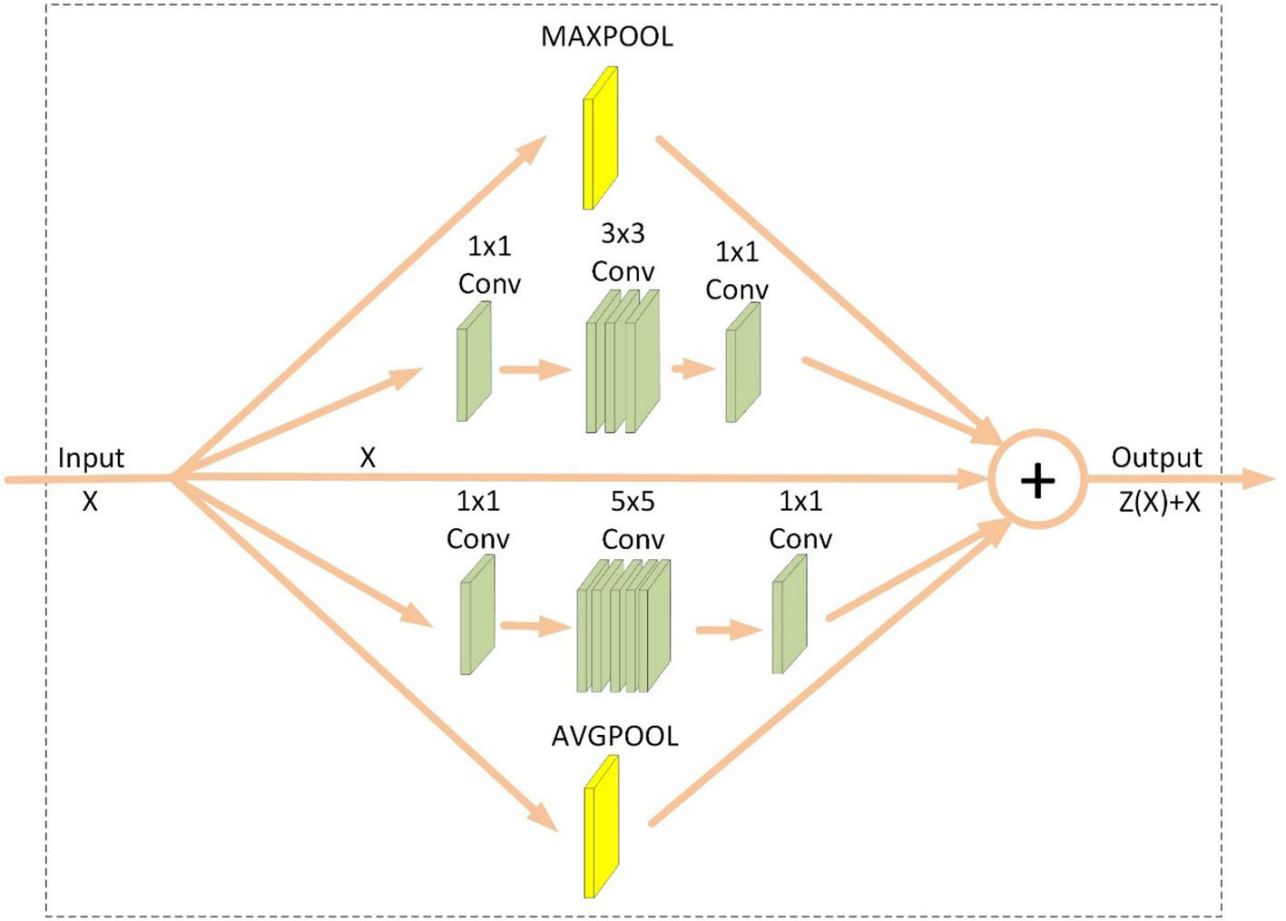
IAP module.

To have an interest in further improving the recognition accuracy of the model, the model is considered to be extended in terms of width, thus the IAP module is proposed. This module adds a layer of MaxPool convolution to Inception V1. To learn more features, the number of channels is upscaled to 128 and 256 dimensions before performing 3x3 and 5x5 convolution and then downscaled to the input dimension size after convolution. The module structure is shown in **Figure 4**, and the parameters of each parallel structure are shown in **Table 2**.

**Table 2:** IAP module configuration.

Module name	Input channels	Output channels	Configure
MaxPool	Input_channel	Output_channel	$\left[\begin{array}{c} Kernel\_size=3\\ Stride=1\\ Padding=1 \end{array}\right]$
3x3Conv	128	128	$\left[\begin{array}{c} Kernel\_size=3\\ Stride=1\\ Padding=1 \end{array}\right]$
5x5Conv	256	256	$\left[\begin{array}{c} Kernel\_size=5\\ Stride=1\\ Padding=2 \end{array}\right]$
AvgPool	Input_channel	Output_channel	$\left[\begin{array}{c} Kernel\_size=3\\ Stride=1\\ Padding=1 \end{array}\right]$

Input_channel = Output_channel, the number of input channels obtained from 1x1Conv before 3x3Conv, 5x5Conv are Input_channel and output channels are 128, 256; the number of input channels obtained from 1x1Conv after 3x3Conv, 5x5Conv are 128, 256 and the number of output channels Output_channel.

The mathematical expression for this module is shown in Equation 1.


(1) $$Z(x)=x+M(x)+C_{3}(x)+C_{5}(x)+A(x)$$

After adding this module to the original model layer3, layer4, and layer5, the features obtained from the shallow layers of the model are fused to improve the recognition accuracy.

### 3.4 MssiapNet

**Figure 5:**
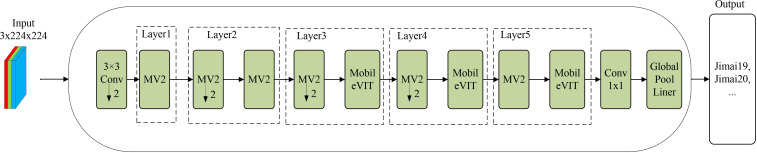
MobileVit-XS.

**Figure 6:**
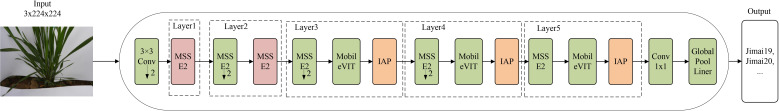
MssiapNet.

The MssiapNet model consists of MSSE2, IAP, and MobileVit modules, and the MobileVit-XS structure is shown in **Figure 5**, and the MagcepNet structure is shown in **Figure 6**.

As shown in **Figure 6**, the attention mechanism and the fusion of multi-channel features are added to the MssiapNet model compared to MobileVit-XS. MssiapNet consists of a 5-layer structure that downsamples the feature map using a 3x3 convolutional block before performing image recognition. Subsequently, feature extraction is performed using MESSE2 blocks and MobileVIT blocks stacked alternately. At the end part of the network, the recognition result of the network is output using Conv and Classifer. The detailed configuration of MssiapNet is shown in **Table 3**.

**Table 3:** Detailed configuration of MssiapNet.

Module name	Output feature map size	Configuration
Conv2d	128×128	$\left[\begin{array}{c} kernelsize=3\\ stride=2 \end{array}\right]$
bn,SiLu	128×128	
MSSE2	128×128	$\begin{array}{l} Conv2d\left[\begin{array}{c} kernelsize=3\\ stride=1\\ padding=1\\ groups=inchannels \end{array}\right]\\ scSE \end{array}$
MSSE2	64×64	$\begin{array}{l} Conv2d\left[kernelsize=1\right]\\ Conv2d\left[\begin{array}{c} \ker nelsize=3\\ stride=1\\ padding=1\\ groups=inchannels \end{array}\right]\\ scSE \end{array}$
MSSE2	64×64	$\begin{array}{l} Conv2d\left[\begin{array}{c} kernelsize=3\\ stride=1\\ padding=1\\ groups=inchannels \end{array}\right]\\ scSE \end{array}$
MSSE2	32×32	$\begin{array}{l} Conv2d\left[kernelsize=1\right]\\ Conv2d\left[\begin{array}{c} kernelsize=3\\ stride=1\\ padding=1\\ groups=inchannels \end{array}\right]\\ scSE \end{array}$
MobileVit×2	32×32	$\left[\begin{array}{c} transformer\_channels=96\\ patch\_h=2,patch\_w=2\\ ffn\_\dim=192\\ num\_heads=4 \end{array}\right]$
IAP	32×32	
MSSE2	16×16	$\begin{array}{l} Conv2d\left[kernelsize=1\right]\\ Conv2d\left[\begin{array}{c} kernelsize=3\\ stride=1\\ padding=1\\ groups=inchannels \end{array}\right]\\ scSE \end{array}$
MobileVit×4	16×16	$\left[\begin{array}{c} transformer\_channels=120\\ patch\_h=2,patch\_w=2\\ ffn\_\dim=240\\ num\_head=4 \end{array}\right]$
IAP	16×16	
MSSE2	8×8	$\begin{array}{l} Conv2d\left[kernelsize=1\right]\\ Conv2d\left[\begin{array}{c} kernelsize=3\\ stride=1\\ padding=1\\ groups=inchannels \end{array}\right]\\ scSE \end{array}$
MobileVit×3	8×8	$\left[\begin{array}{c} transformer\_channels=144\\ patch\_h=2,patch\_w=2\\ ffn\_\dim=288\\ num\_heads=4 \end{array}\right]$
IAP	8×8	
Conv	8×8	kernelsize=1
Classifier	1×1	AdaptiveAvgPool2d,Flatten,Liner

The configuration of the IAP module is detailed in 2.3.3.

### 3.5 Experimental procedure

**Figure 7:**
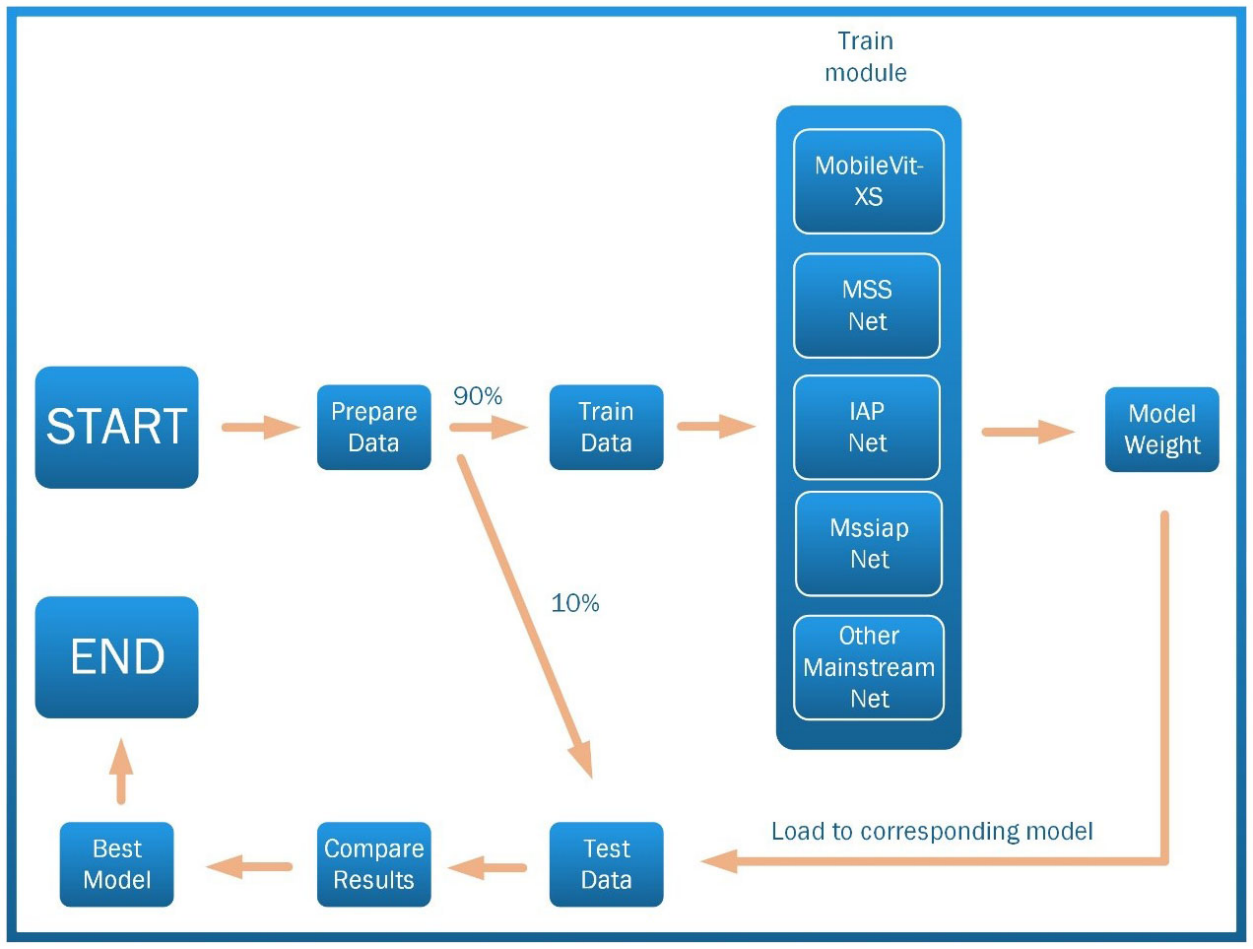
Experimental procedure.

The specific procedure of the experiment is shown in **Figure 7**.

As shown in **Figure 7**, data collection is first performed. The collected data is divided into a training set and a test set in 9:1. Secondly, MobileVit-XS, MSSNET, IAPNET, MssiapNet, and other mainstream networks are trained on the training set. Finally, the model weights obtained from the above models on the training set are loaded to the corresponding models and then tested on the test set, comparing the results obtained to arrive at the optimal model.

### 3.6 Experimental environment

In the local environment, the model is built using the PyTorch framework, the Python version is 3.8, the CPU is Core i5-1135G7, and the graphics card is NVIDIA GeForce MX450. Training with the Dawning Supercomputing Platform of Zhongke Shuguang, the processor used is the HaiGuang 7185, with 128 GB of RAM and a default configuration of 200GB for the compute network.

### 3.7 Experimental details

The hyperparameter settings used for training are shown in **Table 4**.

**Table 4:** Hyperparameter settings for training.

Name	Value	Description
Epochs	400	Number of times the model was trained
Batch size	8	Number of samples selected for one training
Optimizer	SGD	Tool used to bootstrap network update parameters
Learning rate	0.001	Tunes parameters in optimization algorithms
Loss function	Cross Entropy	Evaluates the gap between the predicted value and the true value

### 3.8 Performance evaluation indicators

This experiment’s model performance evaluation metrics include accuracy, precision, recall, F1, number of floating-point operations, specificity, parameters, and heat map after visualization of the model. Accuracy, precision, recall, sensitivity, specificity,and F1 were calculated as shown in Equations 2–6.


(2) $$Accuracy=\frac{TP+TN}{TP+TN+FP+FN}$$

(3) $$\mathrm{Pr}ecision=\frac{TP}{TP+FP}$$

(4) Sensitivity=Recall=TPTP+FN

(5) F1=21Precision+1Recall=2*Precision*RecallPrecision+Recall

(6) $$Specificity=\frac{TN}{TN+FP}$$

TP is the number of positive samples correctly identified; FP is the number of negative samples misreported; TN is the number of negative samples correctly identified; and FN is the number of positive samples missed.

## 4 Experimental results and analyses

### 4.1 Ablation experiments

To investigate whether the accuracy of wheat seedling variety recognition is improved by adding the MSSE2 and IAP modules to the MobileVIT-XS model, the network after adding the MSSE2 module is named MssNet, and the network after adding the IAP module is named IapNet. In the supercomputing platform, 400 rounds of training on the training set using MssNet, IapNet, and MssiapNet, respectively, and the results obtained after testing on the test set are shown in **Tables 5**, **6**.

**Table 5:** Results of ablation experiments.

Models	Influencing factors		Average Accuracy(%)	Average Precision(%)	Flops	Params (MB)	Recall(%)	F1(%)
	MSSE2	IAP						
MssNet	✓	Null	93.86	93.93	7.38x10^8^	8.55	93.88	93.89
IapNet	Null	✓	94.61	94.73	2.63x10^9^	28.57	94.68	94.66
MssiapNet	✓	✓	96.85	96.91	2.63x10^9^	29.70	96.91	96.89

✓ means that the module was applied to the specific model.

**Table 6:** Results of ablation experiments.

Models	Influencing factors		Execution time(s)	Delay time(s)	Specificity(%)
	MSS2	IAP			
MssNet	✓	Null	0.13	0.02	99.74
IapNet	Null	✓	0.15	0.03	99.73
MssiapNet	✓	✓	0.16	0.05	99.83

✓ means that the module was applied to the specific model.

As shown in **Table 5**, after adding the MSSE2 and IAP modules, the recognition accuracy of wheat seedling varieties gradually increases, reaching 93.86%, 94.61%, and 96.85%, but the parameters and floating-point operations also rise. As shown in **Table 6**, the single image execution time of MssiapNet is 0.16s, and the single image latency of MssiapNet is 0.05s, which indicates that the execution time and latency of the model are within the acceptable range.

### 4.2 Model performance exploration

**Figure 8:**
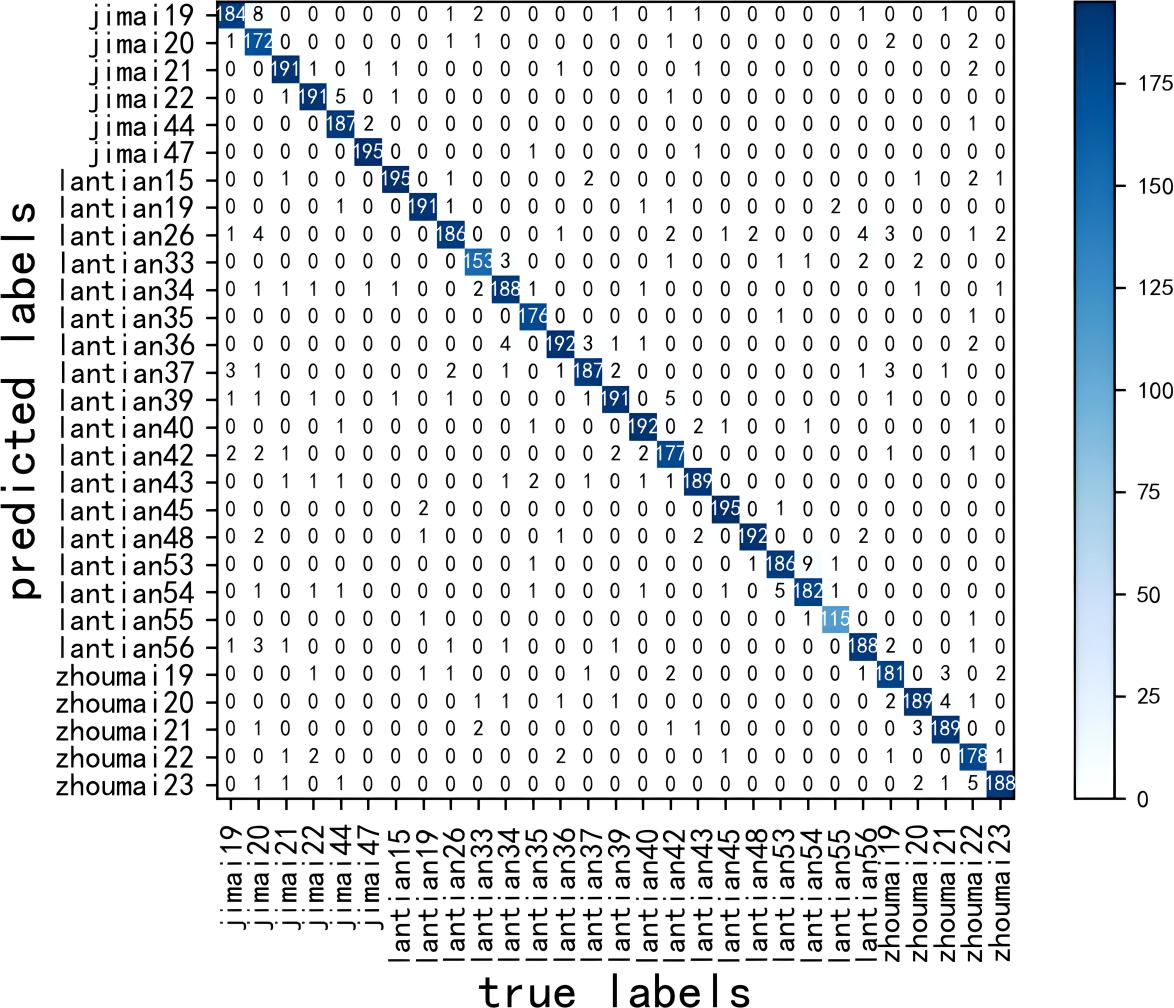
Confusion matrix.

The confusion matrix obtained after MssiapNet was tested on the test set is shown in **Figure 8**, and the precision, recall, and F1 values obtained after recognition of each seedling variety are shown in **Table 7**.

**Table 7:** Identification results of wheat varieties.

Wheat varieties	Accuracy (%)	Recall(%)	F1 (%)	Specificity(%)
jimai19	92.00	95.34	93.64	99.70
jimai20	95.56	87.31	91.25	99.70
jimai21	96.46	95.98	96.22	99.90
jimai22	95.98	95.98	95.98	99.80
jimai44	98.42	94.92	96.64	99.90
jimai47	98.98	97.99	98.48	99.90
lantian15	96.06	97.99	97.02	99.80
lantian19	96.95	97.45	97.20	99.70
lantian26	89.86	95.38	92.54	99.80
lantian33	93.87	95.03	94.45	99.80
lantian34	94.47	94.47	94.47	99.90
lantian35	98.88	96.17	97.51	99.90
lantian36	94.58	96.48	95.52	99.70
lantian37	92.57	95.90	94.21	99.80
lantian39	94.09	95.98	95.03	99.60
lantian40	96.48	96.48	96.48	99.90
lantian42	94.15	91.71	92.91	99.90
lantian43	95.45	95.94	95.69	99.80
lantian45	98.48	97.99	98.23	99.90
lantian48	96.00	98.46	97.21	100.0
lantian53	93.54	95.88	94.90	99.80
lantian54	93.81	93.81	93.81	99.80
lantian55	97.46	96.64	97.05	100.0
lantian56	94.47	94.47	94.47	99.70
zhoumai19	93.78	92.35	93.06	99.90
zhoumai20	94.50	95.45	94.97	99.80
zhoumai21	95.94	94.97	95.45	99.80
zhoumai22	95.70	95.70	95.70	99.90
zhoumai23	94.47	96.41	95.43	99.70

As shown in **Table 7**, after the improved model recognized 29 different varieties of wheat seedlings, except for jimai20, the other varieties’ precision, recall, and F1 values were more than 90%, indicating that the model has a better recognition effect on different wheat seedlings.

### 4.3 Model visualization

**Figure 9:**
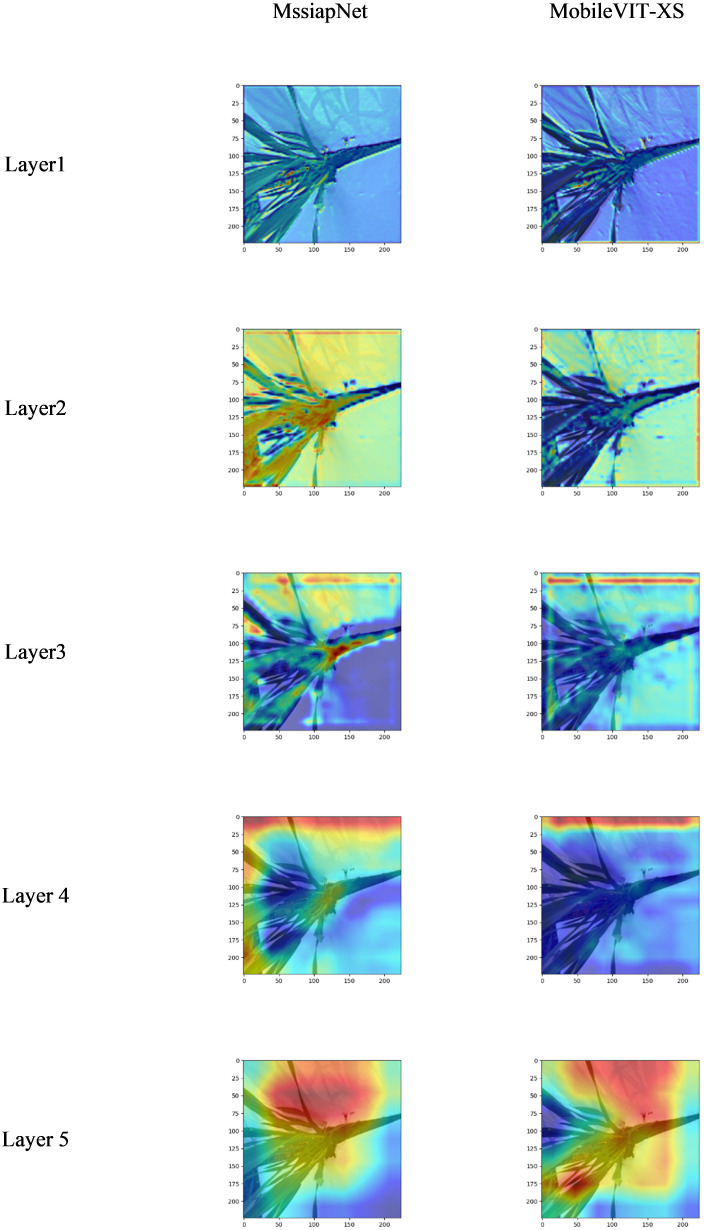
Visualization results.

The heat map obtained by visualizing the original model and the improved model based on the pytorch_grad_cam visualization method is shown in **Figure 9**.

It is clear from the visualization pictures that the improved model focuses more on wheat seedlings and is less disturbed by background information compared to the original model.

### 4.4 Comparison of models

The results after training MobileVIT-XS (Mehta and Rastegari, 2021), MobileNetV2 (Sandler et al., 2018), MobileNetV3 (Howard et al., 2019), EfficientNetV1 (Tan and Le, 2019), EfficientNetV2 (Tan and Le, 2021), ResNet34 (He et al., 2016), RegNet (Radosavovic et al., 2020), ConvNext_tiny (Liu et al., 2022b), ConvNext_small (Liu et al., 2022b) and MssiapNet on the training set for 400 rounds are shown in **Table 8**.

**Table 8:** Comparison of models (the FPS indicator is calculated on the supercomputing platforms).

Models	Average accuracy(%)	Flops	Params(MB)	Recall(%)	FPS(img/s)	F1(%)
MobileVIT-XS	92.17	7.43x10^8^	7.42	92.27	7.87	92.23
MobileNetV2	93.43	3.26×10^8^	13.37	93.41	10.79	93.47
MobileNetV3-large	88.81	**6.11×10^7^**	**5.90**	88.76	10.84	88.85
EfficientNetV1-b0	94.31	4.11×10^8^	15.43	94.26	9.23	94.37
EfficientNetV2-s	95.91	2.89×10^9^	77.11	96.10	4.71	96.07
ResNet34	96.79	3.67×10^9^	83.15	96.82	7.74	96.87
RegNet	74.25	2.07×10^8^	8.87	74.13	**11.12**	74.21
ConvNext-tiny	93.42	4.45×10^9^	106.19	93.54	4.93	93.51
ConvNext-small	94.61	8.68×10^9^	188.69	94.37	3.41	94.45
MssiapNet	**96.85**	2.63x10^9^	29.70	**96.91**	6.64	**96.89**

The meaning of the bolded position indicates that the value is optimal in each comparison network.

As shown in **Table 8**, the bolded parts are the optimal values of each evaluation indicator of the model. Compared to the other nine models, MssiapNet has a higher accuracy of 96.85%. Although the accuracy is only 0.06% higher compared to Resnet34, but parameters are only 1/3 of that. MssiapNet FPS is 6.64img/s, which is relatively low among the compared models, indicating that the model sacrifices some of the FPS to improve the recognition accuracy. RegNet model has the highest FPS of 11.12img/s, but its recognition accuracy is only 74.25%.

## 5 Discussion and conclusions

### 5.1 Discussion

In wheat planting, because of the external environment or some human factors will always lead to wheat planting, such as lack of seedlings, broken ridges, etc. When this happens, wheat growers need to use the same cultivated wheat seedlings as the planted variety to make up for the lack of seedlings and broken ridges to avoid unnecessary economic losses to the growers due to lower wheat yields. Variety identification of wheat seedlings using traditional methods is not suitable for popularisation, although the accuracy of identification is high, the efficiency is low. Deep learning technology allows for rapid identification of wheat seedlings and is simple to operate after mobile phone deployment, making it easy to promote popularity.

A self-constructed wheat seedling dataset, including 29,020 photographs of 29 varieties, was created for this manuscript. Based on this dataset, the MssiapNet model suitable for recognizing wheat seedlings was proposed by improving the lightweight model MobileVIT-XS. Specific areas of improvement are as follows: the scSE module has been added to the original MV2 module to improve the model’s attention to subtle differences between seedlings and to reduce the interference of other information in the identification; A layer of IAP module was added after layer3, layer4 and layer5 of the original module to increase the width of the model and thus improve the accuracy of recognition of wheat seedlings.

The experimental results show that MssiapNet has high recognition accuracy for all varieties of wheat seedlings, with an average recognition accuracy of 96.85%. Highest recognition accuracy compared to other mainstream classification models. After visualizing it, the improved model focuses more on the wheat seedling and is less disturbed by other information than the original model.

The number of MssiapNet parameters is 29.70MB. The single image Execution time of MssiapNet is 0.16s, and the single image Delay time of MssiapNet is 0.05s. The inference speed is fast and meets the needs of daily life, which is convenient for subsequent deployment on mobile to test the model’s actual performance.

### 5.2 Conclusions

This manuscript presents the MssiapNet wheat seedling variety recognition model by improving the MobileVit-XS. After training it on self-constructed wheat seedling data, the average accuracy was 96.85%, the average precision was 96.91%, the recall was 96.91%, and the F1 was 96.89%. Further visualization of the model revealed that compared to the original model, it focused more on wheat seedling information and was less influenced by other factors, such as the background. Compared to MssiapNet with the other nine mainstream classification models, it has the highest recognition accuracy but requires higher Flops, and has a low FPS, requiring further step improvements. The MssiapNet parameter is 29.70MB, which uses a small number of parameters but has a high recognition accuracy. The execution time and Delay time for a single image are 0.16s and 0.05s, which meets the requirements of practical applications deployed on mobile phones.

## Data availability statement

The raw data supporting the conclusions of this article will be made available by the authors, without undue reservation.

## Author contributions

YF: Methodology, Software, Validation, Visualization, Writing – original draft, Writing – review & editing. CL: Funding acquisition, Project administration, Supervision, Writing – review & editing. JH: Funding acquisition, Resources, Supervision, Validation, Writing – review & editing. QL: Resources, Validation, Visualization, Writing – review & editing. XX: Methodology, Software, Supervision, Writing – review & editing.
